# Determinants of the Final Size and Case Rate of Nosocomial Outbreaks

**DOI:** 10.1371/journal.pone.0138216

**Published:** 2015-09-15

**Authors:** Amy Hurford, Alice L. Lin, Jianhong Wu

**Affiliations:** 1 Biology Department, Memorial University of Newfoundland, St John’s, Newfoundland, Canada; 2 Mathematics and Statistics Department, Memorial University of Newfoundland, St John’s, Newfoundland, Canada; 3 Life Sciences Program, Queen’s University, Kingston, Ontario, Canada; 4 Centre for Disease Modelling, York Institute for Health Research, Department of Mathematics and Statistics, York University, Toronto, Ontario, Canada; Columbia University, UNITED STATES

## Abstract

Different nosocomial pathogen species have varying infectivity and durations of infectiousness, while the transmission route determines the contact rate between pathogens and susceptible patients. To determine if the pathogen species and transmission route affects the size and spread of outbreaks, we perform a meta-analysis that examines data from 933 outbreaks of hospital-acquired infection representing 14 pathogen species and 8 transmission routes. We find that the mean number of cases in an outbreak is best predicted by the pathogen species and the mean number of cases per day is best predicted by the species-transmission route combination. Our fitted model predicts the largest mean number of cases for *Salmonella* outbreaks (22.3) and the smallest mean number of cases for *Streptococci* outbreaks (8.5). The largest mean number of cases per day occurs during *Salmonella* outbreaks spread via the environment (0.33) and the smallest occurs for *Legionella* outbreaks spread by multiple transmission routes (0.005). When combined with information on the frequency of outbreaks these findings could inform the design of infection control policies in hospitals.

## Introduction

In 2007, a survey of intensive care units found that the majority of patients were affected by hospital-acquired infections [1]. While hospital-acquired infections are prevalent, different pathogen species are more common and more easily spread [1–4]. For infectious diseases, the transmission rate is affected by characteristics of the pathogen [5], the environment [6,7] and susceptible [1,8,9] and infected patients [10]. Our analysis examines the affect of the pathogen species and the transmission route on the size and spread of nosocomial outbreaks.

We chose to investigate the effect of the pathogen species on outbreak size and spread because different pathogen species have different infectivity, durations of host colonization and survivorship rates on surfaces and equipment. The single admission reproduction number, R_A_, is “the average number of secondary cases caused by one primary case when other patients are susceptible during a single hospital admission of the primary case” [11]. Estimated values of R_A_ for different strains of methicillin-resistant *Staphylococcus aureus* range from 0.07 to 0.93 [12–15] and for different strains of *Acinetobacter baumanii* range from 0.014 to 0.81 [16]. The basic reproductive ratio, R_0_, is “the expected number of secondary cases produced, in a completely susceptible population, by a typical infected individual during its entire period of infectiousness” [17]. For hospital-acquired pathogens, the definitions of R_A_ and R_0_ differ because a colonized patient may be discharged and readmitted while still infectious. The estimated value of R_0_ for vancomycin-resistant enterococci (VRE) is 3.81 [18], and for *Clostridium difficile* is 1.04 [19]. This variation in R_A_ and R_0_ for different pathogen species suggests differences in the spread rates, but in addition to the pathogen strain and species these differences are likely due to the type and geographic location of the health care institution, the infection control measures that were implemented, and the models and parameter estimation methods that were used.

We also focus on the different transmission routes of an outbreak. Many of the pathogen species that cause nosocomial outbreaks may be spread by at least four different transmission routes [4]. Most frequently, hospital-acquired infections are spread between patients or from hospital personnel to patients [4]. Potentially, the transmission route of an infection could affect the size and the spread rate of an outbreak. As an example, if a contaminated piece of equipment is used infrequently, an outbreak spread by equipment might spread more slowly than an outbreak spread by healthcare workers. During a single outbreak, multiple transmission routes may spread the infection. Theoretical analysis shows that when outbreaks are spread by multiple transmission routes the total number of cases (i.e., the ‘final size’ of the outbreak) is either increased or decreased relative to spread by only one of the transmission routes [20].

Infection control priorities could be based on the relative importance of the different transmission routes [21]. The control of large or rapidly spreading outbreaks due to a particular pathogen species might also be prioritized. We analyze published data describing outbreaks of hospital-acquired infection due to 14 different pathogen species spread through 8 possible transmission routes to determine the effect on the total number of cases and the mean number of cases per day.

## Materials and Methods

### Data collection

We compiled data from 1436 nosocomial outbreaks of hospital-acquired infections recorded in the published academic literature using the Worldwide Database for Nosocomial Infections (WDNI; http://www.outbreak-database.com). The recorded outbreaks occurred from 1956 to 2011. Of these outbreaks, only ones with reported transmission routes could be used for our analysis (*n* = 933). We considered outbreaks due to one of 14 different pathogens: *Acinetobacter* sp. (Acin; *n* = 102), *Clostridium difficile* (C. diff; *n* = 15), *Enterobacter* (Enterob; *n* = 46), *Enterococcus* sp. (Enteroc; *n* = 53), *Escherichia coli* (E. coli; *n* = 39), Hepatitis B virus (Hep. B; *n* = 85), Hepatitis C virus (Hep. C; n = 85), *Legionella* sp. (Legion; *n* = 55), *Klebsiella* sp. (Klebs; *n* = 79), *Pseudomonas* sp. (Pseudo; *n* = 144), *Serratia* sp. (Serr; *n* = 116), *Salmonella* sp. (Salm; *n* = 78), *Staphylococcus aureus* (Staph; *n* = 268) and *Streptococcus* sp. (Strep; *n* = 87). These pathogen species were chosen because they frequently cause outbreaks in hospitals and intensive care units worldwide [1] and because for most of these pathogen species, the frequency of outbreaks occurring via each transmission route has been previously studied [4]. For our data analysis, we only considered outbreaks with a single species listed in the ‘Microorganisms’ category of the WDNI.

The transmission routes for the recorded nosocomial outbreaks were air (*n* = 13), environment (env; *n* = 164), equipment (equip; including drugs or biologics; *n* = 267), food (*n* = 30), healthcare workers (HCW; *n* = 296), insect (*n* = 1), patient-to-patient (PTP; *n* = 42), or multiple (multi; *n* = 120), if transmission occurred through more than one route. Full details of the number of observations for each transmission route and pathogen species combination are summarized in Table 1. The designation of a particular transmission route was made if there was a strong statement describing how the infection had spread in the categories ‘Sources’ or ‘Transmission’ of the WDNI or in the abstract of the published record. The difference between the environment and the equipment transmission routes was defined by whether the contaminated article was immovable (environment) or not (equipment).

**Table 1:** The number of observed outbreaks for each transmission route and pathogen species. **In total there are 69 different observed combinations of pathogen species and transmission routes**.

	Transmission route								
Species	air	env	equip	food	HCW	insect	PTP	multi	Total
Acin		20	33		11		7	31	102
C. diff		1	3		4		3	4	15
Enterob		6	24	3	6		2	5	46
Enteroc		20	4		22			6	52
E. coli		1		6	10		6	2	25
Hep B			32		19		5	12	68
Hep C		4	48		11		5	5	73
Klebs		5	18	1	14	1		3	42
Legion		50						1	51
Pseudo		30	48		21			9	108
Salm		5	2	19	11		7	13	57
Staph	4	10	9		119			11	153
Strep	8	6	5	1	28		7	2	57
Serr	1	6	41		20			16	84
Total	13	164	267	30	296	1	42	120	933

Our database recorded whether molecular evidence supported the suspicion of a particular transmission route (i.e., the outbreak strain was also found on healthcare workers, equipment or surfaces); however, too few outbreaks had transmission routes confirmed by molecular data (*n* = 626) for this to be included as a variable in the statistical analysis. For each outbreak, the duration was assumed to be inclusive of the starting and ending months as indicated by the WDNI. Duplicate records and records where the number of cases or the duration was not known were excluded from our analysis. The complete dataset used for our analysis is provided as Supporting Information (S1 Dataset).

### Analyzing the outbreak data

Our statistical analysis tests for differences in the number of cases (CASES) or the mean number of cases per day during an outbreak (the case rate, RATE) due to different pathogen species (SPECIES) and/or transmission routes (TR). The rationale for these choices of response variables (CASES and RATE) is that infection control experts may wish to prioritize the prevention of outbreaks that lead to a large number of cases or that lead to large outbreaks occurring over a small amount of time. The predictor variables are SPECIES, TR and SPECIES×TR.

Our analysis considers 8 models (Table 2) and uses an information theoretic model selection framework to identify the best model(s). Four of our models have CASES and 4 have RATE as the response variable. For each response variable, the models we consider are: constant only, transmission route or pathogen species as a predictor variable, and an interaction model that has specific combinations of transmission routes and pathogen species as the predictor variable. We chose to use an information theoretic framework because we wish to identify the best model(s) from within a set of 4 models, rather than to determine whether specific models are distinguishable from a null hypothesis. All of our models are linear with normally distributed deviations. The predictor variables are coded as 1 if the transmission route and/or pathogen species for an outbreak is equal to *i* or *j* (see Table 1) or 0 otherwise. The distributions of the response variables are both skewed right. We took the natural logarithm of each response variable so that the residuals of our fitted models satisfied our assumption that they were normally distributed.

**Table 2:** The statistical models. **The symbols μ and σ** ^**2**^ **denote the mean and the variance of the normal distribution**. **In this notation *a*, *b***_**i**_, ***b***_**ij**_ **and σ** ^**2**^ **are general model parameters that will take different values for the fitted models**.

Model	Formula
Constant—Cases	log CASES ~ Normal(μ = *a*; σ^2^)
Transmission—Cases	log CASES ~ Normal(μ = Σ *b*_i_ TR_i_; σ^2^)
Species—Cases	log CASES ~ Normal(μ = Σ *b*_i_ SPECIES_i_; σ^2^)
Interaction—Cases	log CASES ~ Normal(μ = Σ *b*_ij_ SPECIES × TR_ij_; σ^2^)
Constant—Rate	log RATE ~ Normal(μ = *a*; σ^2^)
Transmission—Rate	log RATE ~ Normal(μ = Σ *b*_i_ TR_i_; σ^2^),
Species—Rate	log RATE ~ Normal(μ = Σ *b*_i_ SPECIES_i_; σ^2^),
Interaction—Rate	log RATE ~ Normal(μ = Σ *b*_ij_ SPECIES × TR_ij_; σ^2^),

We use the Akaike Information Criteria (AIC) to identify whether the models differ in the ability of their predictor variables to explain the responses. The ΔAIC values were calculated as the difference between a particular model AIC and the lowest AIC from amongst the 4 model set. The ΔAIC values are interpreted as “the models are no different” (ΔAIC < 2), “the models are clearly distinguishable” (4 < ΔAIC < 7) or “the models are definitely different” (ΔAIC > 10, Bolker [22], pp. 210). Having identified the model(s) that best describe the data, we estimate the 95% confidence intervals on the estimated parameters using the maximum likelihood profile. We identify model coefficients that are significantly different from zero at the α = 0.05 level. The significance test assumes that the model coefficients are sampled from a normal distribution. The model analysis was implemented in R (The R Foundation for Statistical Computing, 2013; version 3.02 “Frisbee Sailing”) using the mle2 function from the bbmle package. The code used for this analysis available as Supporting Information (S1 Code).

## Results

### Does the transmission route and/or the pathogen species affect the size and/or rate of spread of the outbreak?

**Fig 1:**
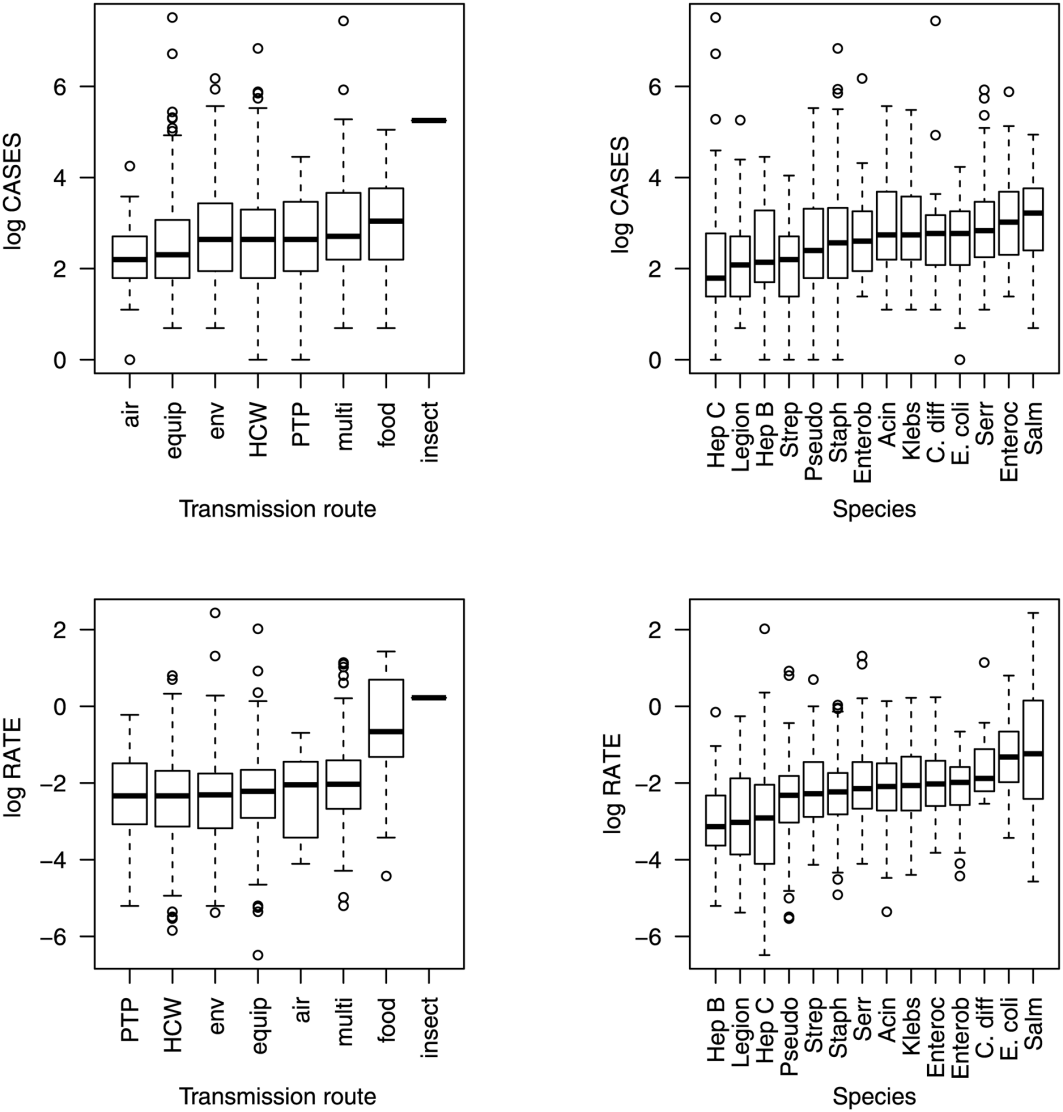
Boxplots showing the natural logarithm of the number of cases and the case rate. The solid lines denote the mean, the boxes represent the interquartile range, the whiskers are 1.5 times the interquartile range, and the open circles are outliers.

**Fig 2:**
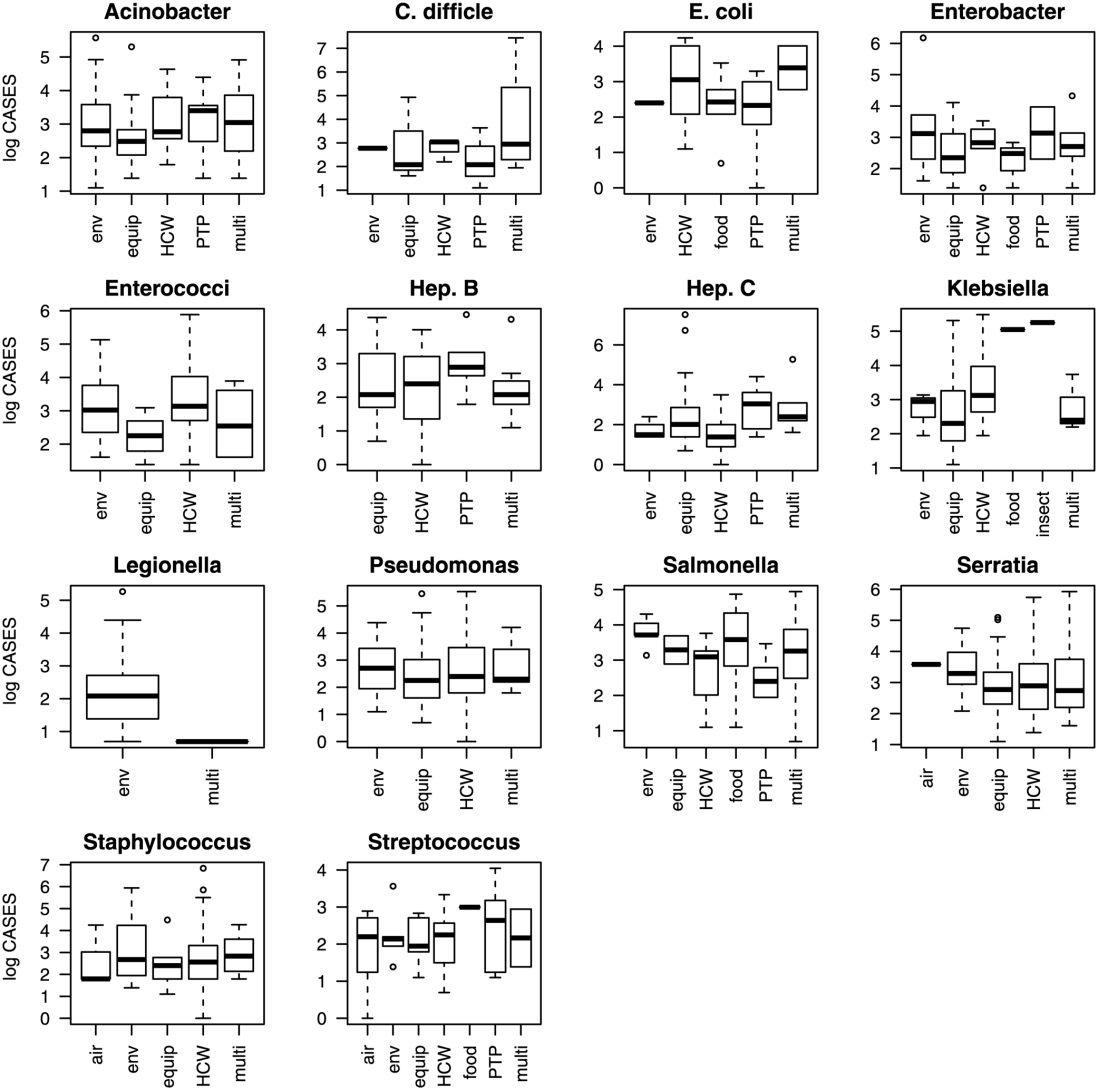
A boxplot showing the natural logarithm of the number of cases for different pathogen species. The solid lines denote the mean, the boxes represent the interquartile range, the whiskers are 1.5 times the interquartile range, and the open circles are outliers. All the coefficients for the Interaction—Cases model are significantly different from zero (α = 0.05).

**Fig 3:**
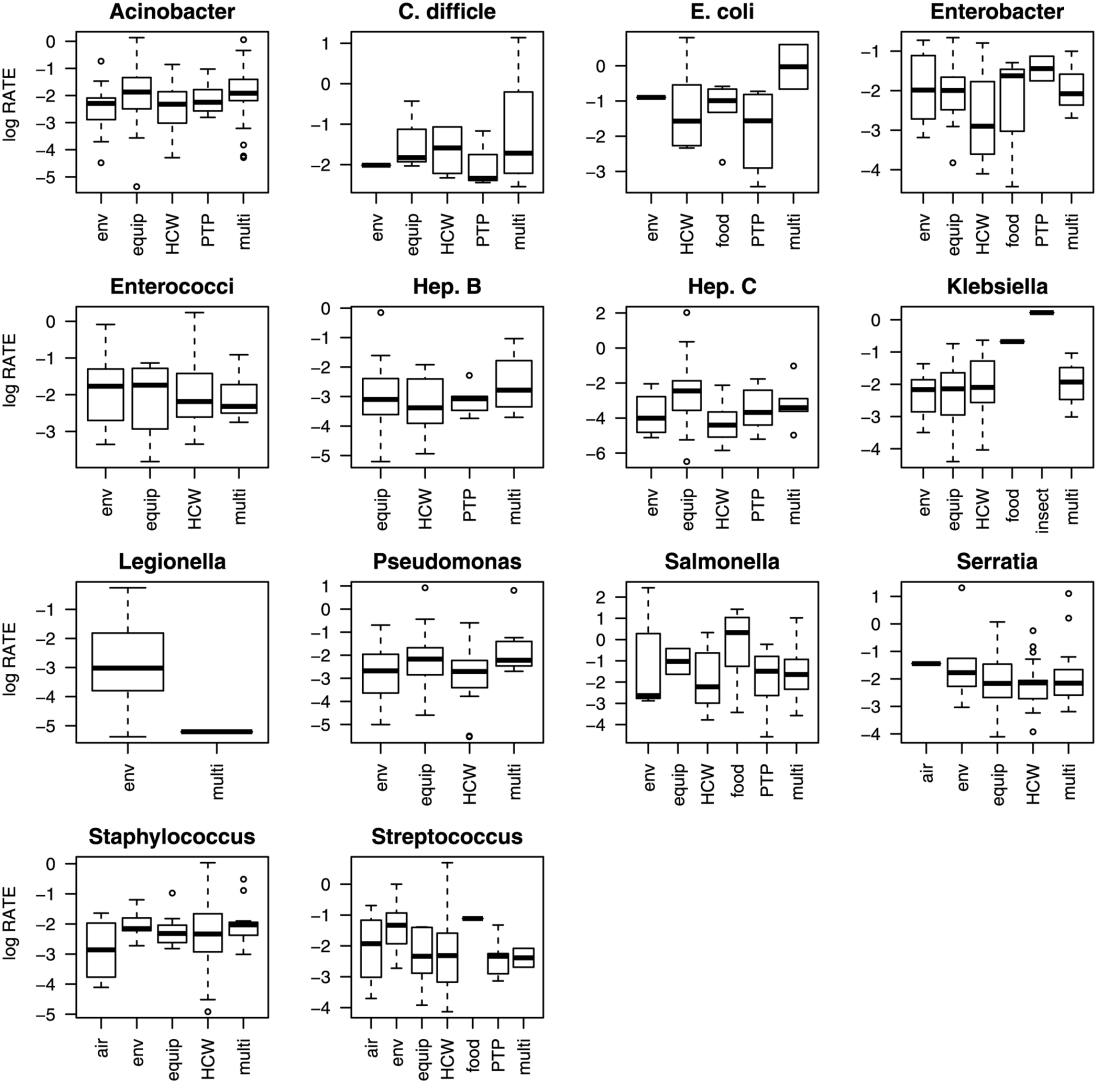
A boxplot showing the natural logarithm of the case rate for different pathogen species. The solid lines denote the mean, the boxes represent the interquartile range, the whiskers are 1.5 times the interquartile range, and the open circles are outliers. The coefficients estimated for the Interaction—Rate model are shown in Fig 5 (lower panel).

The relationship between the transmission route, the pathogen species, and specific combinations of transmission routes and pathogen species on the natural logarithm of the number of cases and on the natural logarithm of the case rate is shown in Figs 1–3. Figs 1–3 show a large amount of overlap in the interquartile ranges of the number of cases and the case rate suggesting that a large amount of the variance around the means of the response variables is not explained by the predictor variables.

**Fig 4:**
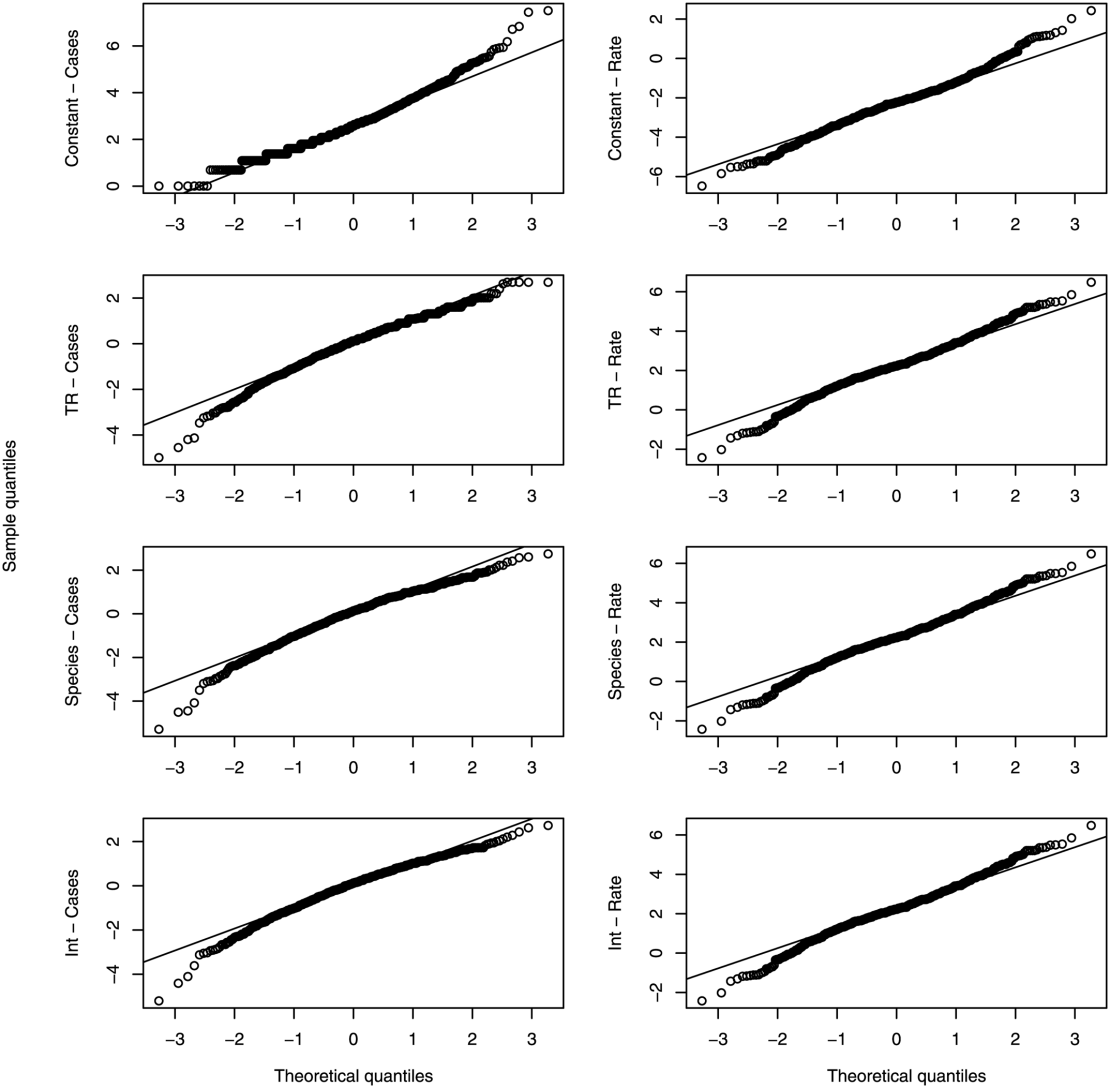
QQ-plots for the natural logarithm of the number of cases and the case rate. The theoretical quantiles are given by a normal distribution. This figure shows good agreement between the data and the assumption that model deviations are normally distributed.

The model where the pathogen species predicts the number of cases (Species—Cases) had the lowest AIC score (Table 3). The ΔAIC for all the other models with CASES as a response variable was greater than 10. The interpretation of this result is that pathogen species is the best predictor of the number of cases in an outbreak, with no other models performing as well. The model with an interaction between the transmission route and the pathogen species improved the model fit, but also introduced numerous parameters resulting in a low AIC score. For the set of models with the case rate as the response variable, the Interaction—Rate model had the lowest AIC score, and the only model with a ΔAIC < 10 was the Species—Rate model (ΔAIC = 5.6, Table 3). A Q-Q plot was used to visualize the comparison between the quantiles of a normal distribution and the quantiles of the model residuals (Fig 4). Fig 4 shows that the assumption of normality of the model residuals is satisfied for all models.

**Table 3:** The AIC values for each model. **For a description of the different models see Table 2**.

Model	Negative log likelihood	Number of Parameters	AIC	ΔAIC	AIC weights
Constant—Cases	1431.65	1	2865.3	64.7	<0.001
Transmission—Cases	1417.76	8	2851.5	50.9	<0.001
Species—Cases	1386.29	13	2800.6	0.0	1.00
Interaction—Cases	1352.99	69	2844.0	43.4	<0.001
Constant—Rate	1531.21	1	3064.4	204.8	<0.001
Transmission—Rate	1480.98	8	2978.0	118.3	<0.001
Species—Rate	1418.61	13	2865.2	5.6	0.05
Interaction—Rate	1360.81	69	2859.6	0.0	0.95

### Which species result in the largest outbreaks?

**Fig 5:**
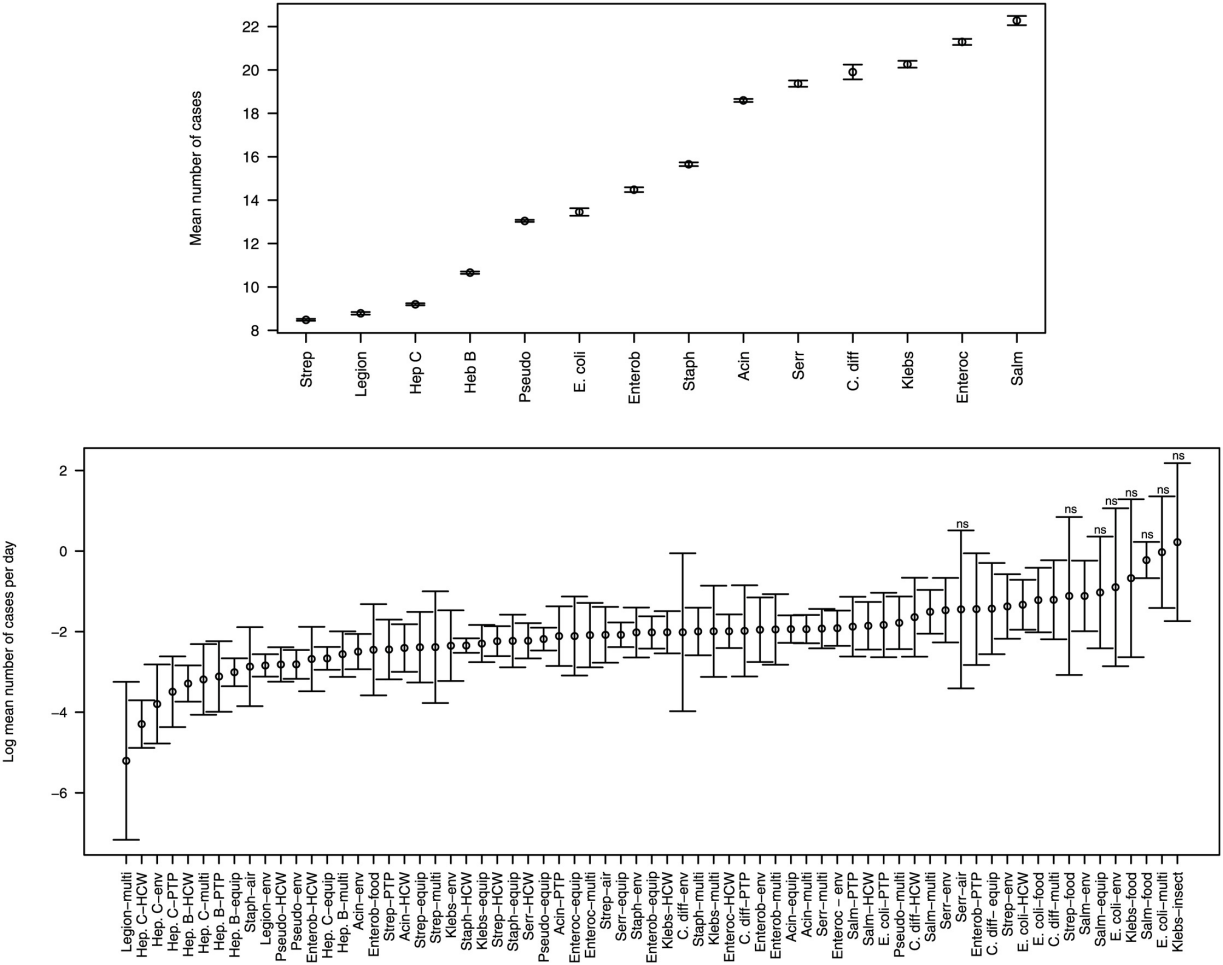
Exponentiated fitted parameter values (circles) and 95% confidence intervals (bars). In the upper panel, the parameter estimates were exponentiated so that the parameter values correspond to the predicted mean number of cases. Models have parameters *b*_*i*_ (see Table 2) where *i* is an index corresponding to a particular pathogen species or transmission route or *b*_*ij*_ where *ij* is an index for a particular transmission route, *i*, and species, *j*, combination. In the lower panel, transmission route-species combinations where the model coefficients are not significantly different from zero are indicated with ‘ns’ (α = 0.05).

For the ‘Species—Cases’ model, the 95% confidence intervals for the exponentiated fitted parameter estimates are shown in Fig 5 (upper panel). The parameter estimates were exponentiated so that the fitted values could be interpreted as the predicted mean number of cases. Fig 5 shows that the confidence intervals on the predicted mean number of cases for each pathogen species are small. For our fitted model, hospital-acquired infections due to *Streptococcus* infections generated the fewest predicted mean number of cases (8.5) while *Salmonella* infections generated the largest predicted mean number of cases (22.3). These *Salmonella* outbreaks were atypical since food was their most common transmission route and food was rarely a transmission route for the other hospital-acquired pathogens (Table 1). Outbreaks of *Enterococcus* sp. caused the second largest number of infections—still more than twice as many as *Streptococcus* sp. and these outbreaks of Enterococci were spread primarily through the healthcare worker and the environmental transmission routes, transmission routes that were common to many of the other pathogen species.

### Which transmission route-species combinations result in the fastest spreading outbreaks?

For the ‘Interaction—Rate’ model, the 95% confidence intervals for the fitted parameter estimates are shown in Fig 5 (lower panel). For this model, the coefficients were not exponentiated for clearer visualization of large and small case rate values. For several of the transmission route-species combinations that had large case rates and small sample sizes, the estimated model coefficients were not significantly different from zero (Fig 5). Of the estimated model coefficients that were significant, the largest predicted mean number of cases per day was for *Salmonella* spread via the environment (0.33), *C*. *difficile* spread via multiple routes (0.30), and *E*. *coli* spread via food (0.30). The smallest predicted mean number of cases per day was for *Legionella* spread through multiple routes (0.005) and Hepatitis C spread through healthcare workers (0.014), the environment (0.022), and person-to-person spread (0.030).

## Discussion

The prevention and control of hospital-acquired infections is a top priority for healthcare institutions [23] and a better understanding of pathogen transmission routes would be beneficial; particularly as infection control strategies could be based on the relative importance of the different transmission routes [21]. Alternatively, prioritization might focus on a particular pathogen species. We found that if preventing large outbreaks is a priority for an infection control strategy, then particular pathogen species should be prioritized from right to left as shown on the horizontal axis of Fig 5 (upper panel). If preventing large outbreaks that occur during a small amount of time is a priority, then particular transmission route-species combinations should be prioritized in order from right to left of Fig 5 (lower panel).

Minimizing the number of cases and the number of cases per day during an outbreak should not be the sole priorities of infection control policies. In particular, the frequency of outbreaks due to particular pathogens and transmission routes is relevant. From Gastmeier et al. [4], the percentage of nosocomial outbreaks caused by different pathogen species is unequal (χ_11_^2^ = 514.6, *p* < 0.001). Most often, outbreaks were due to *Staphylococcus* sp. and *Staphylococcus* outbreaks occurred nearly twice as often as other outbreaks. The conclusions that can be drawn from our data are the same (Table 1; S1 Code). Other published studies consider the point prevalence of different pathogen species in intensive care units [1], the number of cases in intensive care units [8] and the number of cases of bloodstream infections [2] caused by different pathogen species. These studies also find that *Staphylococcus* sp. infections are most common, although in the intensive care unit *Pseudomonas* infections are nearly as common [1,8]. These findings may be a reason to prioritize the prevention of *Staphylococcus* outbreaks, although the severity of the disease, the cost and effectiveness of potential infection control measures, and institution specific surveillance are other factors that should be considered when devising an optimal infection control strategy.

Limitations of our data analysis are potentially misidentified transmission routes, publication biases, possible inconsistencies in how an epidemic is defined, and a large amount of unexplained variation. Quantifying the number and magnitude of the transmission routes that contribute to an outbreak is challenging [24]. In our dataset outbreaks that were designated as having been spread by one transmission route may have actually been spread through multiple transmission routes, where the additional transmission routes were not identified. We designated an outbreak as having occurred through a particular transmission route if there was “a strong statement describing how the infection had spread” in the WDNI or in the abstract of the published record. As such, the evidence to support the designation of a transmission route was of variable quality and potentially could have been based on erroneous designations from the published record. Our analysis of nosocomial outbreaks was based on published accounts of outbreaks, which could be misrepresentative of outbreaks in general. There is a geographic bias in the assembled data since most published outbreaks are from Europe or North America, while hospital-acquired infections occur worldwide [1]. There may be a publication bias if data from large outbreaks are more likely to be submitted for publication. The individual authors of each published report judged when the end of an outbreak occurred. Some reported outbreaks were very long, having lasted more than a year, and a possible inconsistency could arise if other authors would have judged the same outbreak to be several smaller outbreaks. Even our best models failed to explain a large amount of variation in the number of cases. Nosocomial outbreaks are influenced by many factors, but our analysis considered only pathogen species and transmission routes. Other relevant factors, and potential sources of variation, are the type of healthcare institution [6], and whether the patient is intubated, ventilated or has a catheter or central line [8].

Our analysis finds that the pathogen species is the best predictor of the number of cases and the transmission route-species combination is the best predictor of the number of cases per day during an outbreak. These findings will help to inform priorities for infection control strategies.

## Supporting Information

S1 Code R computer code to analyze the data file. (R)

S1 Dataset Data files in Microsoft Excel and CSV formats. (ZIP)
